# Hunger influenced life expectancy in war-torn Sub-Saharan African countries

**DOI:** 10.1186/s41043-018-0143-3

**Published:** 2018-04-27

**Authors:** Florence N. Uchendu

**Affiliations:** Faculty of Health Sciences, Department of Public and Environmental Health Sciences, National Open University of Nigeria, University Village, Plot 91, Cadastral Zone, Nnamdi Azikiwe Express Way,, Jabi-Abuja, Nigeria

**Keywords:** Malnutrition, War, Hunger, Undernourished, Food security, Life expectancy, Sub-Saharan, Developing countries

## Abstract

**Background:**

Malnutrition is a global public health problem especially in developing countries experiencing war/conflicts. War might be one of the socio-political factors influencing malnutrition in Sub-Saharan African (SSA) countries. This study aims at determining the influence of war on corruption, population (POP), number of population malnourished (NPU), food security and life expectancy (LE) in war-torn SSA countries (WTSSA) by comparing their malnutrition indicators.

**Methods:**

Fourteen countries in WTSSA were stratified into zones according to war incidences. Countries’ secondary data on population (POP), NPU, Food Security Index (FSI), corruption perceptions index (CPI), Global Hunger Index (GHI) and LE were obtained from global published data. *T* test, multivariate and Pearson correlation analyses were performed to determine the relationship between CPI, POP, GHI, FSI, NPU, male LE (MLE) and female LE (FLE) in WTSSA at *p* < .05. Data were presented in tables, means, standard deviation and percentages.

**Results:**

Mean NPU, CPI, GHI, POP, FSI, MLE and FLE in WTSSA were 5.0 million, 28.3%, 18.2%, 33.8 million, 30.8%, 54.7 years and 57.1 years, respectively. GHI significantly influenced LE in both male and female POP in WTSSA. NPU, CPI, FSI, GHI and FLE were not significantly different according to zones except in MLE.

**Conclusions:**

Malnutrition indicators were similarly affected in WTSSA. Hunger influenced life expectancy. Policies promoting good governance, equity, peaceful co-existence, respect for human right and adequate food supply will aid malnutrition eradication and prevent war occurrences in Sub-Saharan African countries.

## Introduction

Malnutrition is a global public health problem in Sub-Saharan Africa (SSA) [1–3]. One of the serious forms of malnutrition is hidden hunger. Globally, an estimated two billion people are affected by deficiencies associated with hidden hunger and one in three people suffer from it [1, 2]. Sub-Saharan Africa has high prevalence of hidden hunger [2–4]. Micronutrient deficiencies affect many people of all genders and ages including some at-risk groups such as pre-school children, pregnant and lactating mothers and adolescents. Examples of micronutrient malnutrition are vitamin A deficiency (VAD), iodine deficiency disorder (IDD), iron deficiency and anaemia (IDA), zinc deficiency and folic acid deficiency. Nigeria has been experiencing one form of conflict or the other in the past few years, for example ‘Boko Haram’, ‘Niger delta avengers’, 'herds men' and ‘Biafra’ thereby displacing many citizens. In Nigeria, 29.5% pre-school children are vitamin A deficient. About 75% are iron deficient and anaemic. Micronutrient malnutrition causes night blindness, poor physical growth, poor development, low intelligent quotient, low intellectual and cognitive abilities, neural bifida and thyroid dysfunction, mental impairment and poor health and productivity including death.

War and conflict are rampant in many developing countries especially in SSA due to conflicts over basic resources such as food and water, access and control over rich minerals, unresolved political issues and agendas, effect of global financial crisis, rising costs of living, human rights violations, land and border disputes, harsh dictatorship, high unemployment especially among educated youths, frustration from decades of living under authoritarian and corrupt leaders and environmental degradation as reported by Goldson [5] and Shah [6]. Corrupt practices have been reported to negatively influence food security and life expectancy in developing countries [7]. Poverty, hunger and gross food insecurity are ravaging the masses not because of lack of resources in some cases but due to the absence of a ‘messiah’ or a true patriot or selfless advisors and managers of national resources and tax payers’ money [8]. Indirect consequences of war include the disruption of health care and education, high prevalence of gastrointestinal and respiratory infections (pneumonia, tuberculosis, dysentery and cardiovascular diseases), malnutrition, maternal under-nutrition, increased number of refugees and childhood mortality and morbidity. During war, there is scarcity of agricultural and social amenities resulting in rationing of foods and water thereby aggravating chronic hunger and starvation. Malnutrition already existing in Africa is exacerbated under war conditions with resulting increased mortality and morbidity [5].

One of the most significant consequences of war is the number of refugees. There have been over nine million refugees and internally displaced people from conflicts in Sub-Saharan Africa [6]. During the Vietnam war, the enormous number of refugees resulted in sparse medical equipment and inadequate maintenance capability with only 150 Vietnamese doctors available to care for 15 million civilians. Consequently, life expectancy was approximately 35 years, half the children born died before their fifth birthday and the infant mortality was estimated to be 225 per 1000 live births. In Liberia, pre-conflict acute malnutrition was 1.6%, while during the onset of the conflict which started in December 1989, malnutrition prevalence rose between 10 and 50% [9]. Increase in malnutrition periodically upsurged according to the scale of conflict and displacement of segments of the population. Similar trends were found in Somalia, Sudan and many other countries [9].

Few studies have compared the influence of war on malnutrition indicators such as food security, life expectancy, number of population undernourished and hidden hunger in developing countries especially those experiencing one form of war or conflict to ascertain the actual relationship between these indicators. This study aims at bridging this gap.

## Methods

### Inclusion criteria

Only countries experiencing war/conflict in SSA were selected. Countries were excluded if they do not have complete relevant data (CPI, FSI, POP, GHI, NPU, LE, MLE and FLE) available. Global Food Security Index (GFSI) 2013 and 2014 only had records for 107 and 109 countries, respectively. Many developing countries do not have GFSI and GHI data. Only 14 out of 26 countries experiencing war or conflicts in SSA that had complete relevant data available were included.

### Data collection

The researcher surfed the net to identify ‘SSA countries undergoing war or some form of conflicts’. Stratified sampling technique was used in selecting countries based on incidence of war. Sub-Saharan African countries experiencing war with their socio-economic and malnutrition indices were grouped into zones: West Africa, East Africa, Central Africa and North Africa. Out of 26 SSA countries experiencing war, only 14 with complete relevant data were purposely selected. Qualitative and quantitative data were collected. Secondary data on countries’ corruption perceptions index (CPI), Global Food Security Index (GFSI), population (POP), Global Hunger Index (GHI), number of population undernourished (NPU) and life expectancy (LE) (male and female) scores were collected from published indexes [10–14].

### Data analysis

Data was collected and captured in an Excel sheet. *T* test, multivariate regression (Wilks’ lambda) and Pearson correlation analyses were done to determine the relationship between GHI, CPI, GFSI, POP, NPU and LE in war-torn countries in SSA in zones. Data were presented in tables, means, standard deviation and percentages.

## Result

Table 1 summarised WTSSA countries and their socio-economic and malnutrition indices in zones. Mean value for malnutrition indicators in WTSSA countries were NPU 5.0 million, CPI 18.2, population 33.8 million, GFSI 30.8, MLE 54.7 years and FLE 57.2 years. Table 2 shows the relationship between GHI, CPI, GFSI, POP, NPU and LE in WTSSA. There was no significant relationship between CPI, GFSI, POP, NPU and GHI in WTSSA (*p* > .05). But GHI influenced life expectancy in both male and female populations (*p* < .05). Also, FLE was significantly different from MLE (*p* < .05). Table 3 compares GHI, CPI, GFSI, POP, NPU, MLE and FLE in WTSSA by zones. Malnutrition indicators were not significantly different across the zones except in male life expectancy (*p* < .05). War similarly affected all malnutrition indicators in WTSSA as shown in Table 3.

**Table 1:** Sub-Saharan African countries experiencing war and their socio-economic and political indices in zones

S/N	SSA region	Country	NPU (million)^a^	CPI 2013^b^	GHI 2014^c^	POP (million)^d^	GFSI 2013^e^	LE at birth (years)^d^	
								Male	Female
1	West Africa	Cote d’Ivoire	3.0	27	15.7	21.1	39.5	49	51
2		Guinea	2.1	24	14.3	11.8	32	55	56
3		Mali	1.1*	28	13.0	15.5	26.8	52	56
4		Nigeria	11.2	25	14.7	150	33	51	52
5		Senegal	2.4	41	14.4	13.5	34.5	62	65
6		Sierra Leone	1.6	30	22.5	6.2	29.0	45	45
7		Togo	1.0	29	13.9	6.2	22.7	55	57
8	East Africa	Ethiopia	3.9	33	24.4	89.2	31.2	61	64
9		Kenya	10.8	27	16.5	44.2	36.4	59	62
10		Rwanda	4.0	53	15.6	11.1	29.3	61	65
11		Uganda	9.7	26	21.5	36.9	38.3	57	59
12	Central Africa	Angola	3.9	23	17.4	21.6	31.8	50	53
13		Chad	4.5	19	24.9	12.2	22.1	49	51
14	Northern Africa	Sudan	11.4**	11	26.0	34.2	25 ± .2	60	63
Mean			5.0 ± 3.9	28.3 ± 9.8	18.2 ± 4.6	33.8 ± 39.9	30.8 ± 5.4	54.7 ± 5.5	57.1 ± 6.2

^a^FAO, 2014

^b^Transparency International, 2013

^c^IFPRI, 2014

^d^Population Reference Bureau, 2013

^e^The Economic Intelligent Unit Limited, 2013

*2005–2007 data

**2009–2011 data

**Table 2:** Relationship between GHI, CPI, GFSI, NPU and LE in war-torn SSA countries

	GHI	CPI	GFSI	NPU	MLE	FLE
GHI	1					
CPI	− .386	1				
GFSI	− .251	.187	1			
NPU	.325	− .373	.266	1		
MLE	− .004*	.359	.118	.270	1	
FLE	− .022*	.384	.090	.242	.987^*^	1

*p < 0.05

**Table 3:** Comparison of mean GHI, CPI, GFSI, POP, NPU and LE in SSA war-torn countries by zones

	Mean ratings of zones in Africa				
	West	East	Central	*F* ratio	*p* value
GHI	15.5	18.9	21.2	2.384	0.138
CPI	29.1	32.0	21.0	1.157	0.350
GFSI	31.1	33.8	26.9	1.124	0.363
NPU	3.2	6.6	4.2	1.496	0.266
MLE	52.7	58.6	49.5	4.268	0.042*
FLE	54.6	61.2	52.0	3.396	0.071
POP	32.0	38.9	16.9	0.186	0.833

Comparison for North Africa was not conducted because only one country that had complete data was used

*p < .05

## Discussion

Only 53.9% of countries in developing countries experiencing some kind of war or conflicts had complete data. This is an indication that war periods precipitate loss of data which could aid policy decisions. Countries in West Africa experienced the highest incidence of war (50.0%) followed by East Africa (28.6%), Central Africa (14.3%) and then North Africa (7.1%). The high number of population malnourished (5.0 million) in war-torn countries of SSA justifies the low GHI (18.2%) and GFSI (30.8%) as shown in Table 1. During war, people are unable to produce or buy enough food and so depend on humanitarian gestures which were never enough. Chronic hunger and famine is normally prevalent leading to malnutrition across the populace. This agrees with the reports of [5, 6, 8]. War-torn SSA countries also had very low mean CPI, 28.3%. This agrees with [7]. Corrupt practices divert public fund to private use, thereby depriving the poor masses of basic social amenities, and this might be one of the reasons for war. This agrees with the report of [8]. There was a significant relationship between MLE, FLE and GHI in WTSSA countries (*p* < .05) as shown in Table 2.

Chronic hunger results in severe malnutrition which shortens the length of time people could live. Significant difference existed in the MLE across the zones. Central Africa had the least MLE (49.5 years) followed by the Western zone (52.7 years). These zones experience war and conflicts more than the East zone. East Africa had a better mean corruption perception index (32%) even though it is still low, the highest GFSI (34%), MLE (59%) and FLE (62%) (Table 3).

The East zone had the highest number of people under nourishment (NPU 6.6 million) and was the second zone with a higher Global Hunger Index (18.9%), yet the zone scored highest in MLE and FLE indicating that probably in war situations, other factors could be employed to help the citizens to live better healthy lives such as adequate medical care, food supplementation, nutrition education, relief materials from other countries and food diversity using local staples. It is normally during war that new nutritious staples are discovered. East Africa might have had alternative sources of food during the war which made its GFSI the highest among the zones in SSA (33.8%). The significant difference between FLE and MLE might be because males are recruited into war at young ages.

## Conclusion

All the mean values for malnutrition indicators in WTSSA were generally low, below 50%. Malnutrition indicators were similarly affected in WTSSA especially male and female life expectancies. Policies promoting good governance, equity, peaceful coexistence, respect for human right and adequate food supply will help to eradicate malnutrition and prevent war occurrences in Sub-Saharan African countries.

## Abbreviations

CPI: Corruption perceptions index
FAO: Food and Agricultural Organisation
FLE: Female life expectancy
FSI: Food Security Index
GFSI: Global Food Security Index
GHI: Global Hunger Index
IDA: Iron deficiency and anaemia
IDD: Iodine deficiency disorder
IFPR: International Food Policy Research Institute
LE: Life expectancy
MLE: Male life expectancy
NPU: Number of population malnourished
POP: Population
SSA: Sub-Saharan Africa
VAD: Vitamin A deficiency
WTSSA: War-torn Sub-Saharan African countries
